# Consumer Nutritional Awareness, Sustainability Knowledge, and Purchase Intention of Environmentally Friendly Cookies in Croatia, France, and North Macedonia

**DOI:** 10.3390/foods12213932

**Published:** 2023-10-27

**Authors:** Dunja Molnar, Elena Velickova, Carole Prost, Mishela Temkov, Mario Ščetar, Dubravka Novotni

**Affiliations:** 1 University of Zagreb Faculty of Food Technology and Biotechnology, Pierottijeva 6, 10000 Zagreb, Croatia; dunjamolnar151@gmail.com (D.M.); mscetar@pbf.hr (M.Š.); 2 Faculty of Technology and Metallurgy, Ss. Cyril and Methodius University in Skopje, Rudger Boskovic 16, 1000 Skopje, North Macedonia; velickova@tmf.ukim.edu.mk (E.V.); mishela@tmf.ukim.edu.mk (M.T.); 3 ONIRIS, Nantes Université, CNRS, GEPEA, UMR 6144, F-44322 Nantes, France; carole.prost@oniris-nantes.fr

**Keywords:** food byproducts, generational differences, nutritional awareness, sustainability knowledge

## Abstract

The increasing demand for greater utilization of byproducts in the food industry has been driven by growing interest in environmental sustainability. This paper examines the market potential and consumer attitudes toward whole-grain and sustainable cookies made with food byproducts and edible films. Additionally, particular attention was given to evaluating levels of sustainability knowledge and nutritional awareness, willingness to purchase environmentally friendly cookies with food byproducts, and to appraise differences in answers between countries and generations. An online questionnaire was used to collect data from Croatian (*n* = 472), French (*n* = 166), and North Macedonian consumers (*n* = 119) aged between 18 and 62, predominantly women (82%) with higher education degrees. Results showed that even if chocolate-coated cookies remain very popular, North Macedonians prefer whole-grain and plain cookies, while the French prefer chocolate-coated cookies and Croats prefer both types of cookie. The majority of consumers (96%) were interested in purchasing environmentally friendly cookies. However, consumers’ interest in purchasing cookies with food byproducts was generally low, which may be related to their limited knowledge of byproducts. In conclusion, there is market potential for whole-grain cookies with food byproducts, but brand, price, and consumer education may be critical to their success.

## 1. Introduction

Consumers are showing an increasing inclination toward food that offer health benefits and are increasingly interested in the health benefits of food rich in nutrients and bioactive compounds such as antioxidants, polyphenols, fiber, minerals, and vitamins [1,2]. As a result, efforts have been made to develop food that can maintain and improve people’s health [3,4]. Food based on whole grains, which are rich in nutrients, fibers, and phytochemicals, with proven health benefits, play significant role in maintaining human health and wellbeing [5]. Although the amount of whole-grain food on the market is constantly increasing [6], its consumption remains low.

Cookies are a very popular cereal-based food consumed by both children and adults worldwide [7], so they could be used as a suitable vehicle for nutrient incorporation [8]. The highest number of newly developed products can be found in the sweet cookie category, where claims such as high in/source of fiber and whole grain are among the top five used claims [9]. Cookie consumption does not have to be contrary to the healthy indulgence trend and can even be used as part of nutritional strategies to tackle several nutrient deficiencies as well as chronic and nutrition-related diseases [10,11], such as diabetes, celiac disease, obesity, and cardiovascular diseases [12,13,14,15]. On top of this, the sustainability in the supply chain of cocoa as a popular ingredient in cookies has been challenged.

Consumer interest in the environmental sustainability of food production has also increased in recent years [16]. One of the main problems facing the global food system is food waste, as more than one-third of the food produced for human consumption is either lost or wasted each year [17,18,19,20]. In this regard, byproducts from the fruit industry can be an optimal source of bioactive compounds and can serve as value-adding ingredients for cookies [21]. For instance, grape and aronia (chokeberry) pomace could serve as a partial substitute for cocoa powder in cookies [22,23]. Nevertheless, the market for cookies with byproducts as upcycling ingredients is still limited.

Packaging plays an important role in the marketing and shelf life of cookies. The use of edible and biodegradable films could reduce the amount of disposable packaging used and therefore reduce environmental pollution and CO_2_ emissions. These materials should be employed in multilayer packaging, improve organoleptic properties, supplement nutritional value, serve as carriers for antioxidants and microbiological agents, and control the transfer of moisture, gasses, and other compounds in the food system [24]. Edible films based on chitosan and gum arabic enriched with grape seed extract can improve the bioactive profile [12] and extend the shelf life of cookies [24] without affecting product quality or consumer taste.

Based on the above, it could be suggested that the fortification of popular cookies with fruit byproducts and edible films could result in a product that easily delivers important nutrients such as antioxidants, minerals, and fiber to a wider population [12,24]. However, understanding how and why consumers purchase functional or sustainable (upcycled) foods is important for the sustainable development of this sector [20,25]. Previous studies investigated and explained consumer behavior related to purchase intention, acceptance, and consumption of cookies [19,26,27], which depends on the sensory profile of cookies and consumer habits, demographic background, personal relevance of functional benefits [28,29], marketing strategies, and on-pack labeling [30]. Various factors such as availability, quality, brand, novelty, packaging, and pricing have been identified to affect the behavior of cookies consumers. [15,31]. However, only a limited number of studies have investigated the market potential of functional and sustainable cookies.

The aim of this study was to investigate the market potential and consumers attitudes towards whole-grain and sustainable cookies in three Mediterranean countries (Croatia, France, and North Macedonia). Special attention was paid to differences between countries and generations in the preference of cookie type, consumption motivation, nutritional awareness, sustainability knowledge, and willingness to purchase cookies containing food byproducts. Relations between participants responses and their country of residence and generation group were evaluated through analysis of variance, principal component, and cluster analysis. Further on, the importance of sociodemographic factors (education, country of residence, generation, and gender) on willingness to purchase cookies, which are beneficial to health but also the environment or contain food byproducts, was assessed using classification and a regression tree. Our hypothesis was that a purchase intention is positively influenced by consumer education degree and differs between generations and countries.

## 2. Materials and Methods

### 2.1. Study Design and Study Population

This cross-sectional study was conducted using an anonymous online questionnaire created on the Google Forms online survey platform. The questionnaire was created in Croatian and then translated into French and Macedonian. Data collection was conducted in two intervals from April 2022 to June 2023. The research team distributed invitations to participate in this study to their professional networks and personal contacts via e-mail and social networks (Facebook^®^ and LinkedIn^®^). To increase the number of study participants, research team members asked potential participants to forward the invitations to their contacts. Eligible participants were adults above 18 years of age, residents of Croatia, France, or North Macedonia with access to a computer, tablet, or phone with an internet connection. A total of 797 participants (Croatia (*n* = 500), France (*n* = 172), North Macedonia (*n* = 125)) completed the online questionnaire. Participants who responded that they never consume cereal-based foods (*n* = 40) were excluded from further analysis. Based on the study by Šedík et al. [32], participants were divided into four generations as follows: Generation Z (born between 2004 and 1997), Generation Y (born between 1996 and 1981), Generation X (born between 1980 and 1972), and Generation Silver (born between 1971 and 1952). Sociodemographic characteristics of participants who consumed cereals are shown in Table 1. Generation Y was the most strongly represented. Most study participants were female, employed and had a higher level of education (bachelor’s or master’s degree).

All participants were voluntarily enrolled in the study and were assured of strict confidentiality. In the participant information sheet at the beginning of the online questionnaire, potential participants were provided with detailed information about the authors of the study, affiliations, the main research findings, the indication that the results will be used in a doctoral dissertation, and the estimated time required to complete the questionnaire (10–12 min). All participants had the right to withdraw from the study at any time without consequence. There were no adverse health effects that could be caused by participation in the present study. Participants did not receive any financial or other compensation for their participation in the study.

All participants signed an informed consent form to participate in this study. This study was conducted in accordance with the Declaration of Helsinki, and approved by the Ethics Committee of the Food Technology and Biotechnology (University of Zagreb Faculty, Croatia, 251-69-11-20-37; 14 December 2020), the Faculty Committee of the Faculty of Technology and Metallurgy (Ss. Cyril and Methodius University in Skopje, Republic of North Macedonia, 09-1273/1; 10 November 2020), and the ONIRIS institution (Oniris VetAgroBio—Ecole Vétérinaire, Agroalimentaire et de l’Alimentation Nantes Atlantique—French Republic Agricultural Ministry National HighSchool, France, 23-657; 12 February 2023).

The research data were collected using a structured online questionnaire adapted from Čukelj et al. (2016) [33], which consisted of a total of 43 multiple-choice questions divided into several sections: sociodemographics (5 questions), cookie consumption (9 questions), purchase intention (8 questions), brand (4 questions), nutrition and health (9 questions), and sustainability (3 questions).

Sociodemographic information included questions regarding age, gender, education, employment status, and country of residence. In the cereal product consumption section, data regarding the type of products consumed, frequency of consumption, and consumption habits were collected. The cookie consumption section included questions regarding preferred product type, consumption habits (reasons and key drivers for consumption), as well as the frequency of consumption. Data collected in the nutrition and health category focused on reading nutrition labels, and intake of dietary fiber and bioactive compounds. The section on sustainability focused on general knowledge and familiarity with sustainability/edible films and fruit byproducts. Within the purchase intention section, the brand preference for purchasing domestic or foreign producers, the willingness to pay a higher price for modified cookies or buy cookies of improved nutritional value/cookies with food industry byproducts/cookies with positive environmental impact was investigated.

Consumption of cereal products and cookies, as well as food rich in fiber and biologically active compounds, was assessed using a 5-point Likert scale ranging from 1 (never) to 5 (always). Consumer purchase behavior and nutrition awareness were measured using a 5-point Likert scale ranging from 1 (strongly disagree) to 5 (strongly agree). Finally, participants were asked to indicate their willingness to purchase nutritionally enhanced and sustainable products (cookies with byproducts of the food industry) on a 2-point scale from 1 (no) to 2 (yes). Zero was included in these questions as a “do not know” or “would not like to answer” option.

### 2.2. Nutritional Awareness Index and Sustainability Knowledge Score

The index of nutritional awareness (INA) indicates greater understanding or improved application of recent and accepted nutritional principles [33]. It was formed from five questions or statements with loadings of 0.6 or more, which explained 45% of the total variance after factor analysis (Table 2). Questions (“How often do you consume food rich in dietary fiber?”; “Do you introduce biologically active compounds into your body daily?”; “Are you reading food labels (nutritional data and ingredient list)?”) and statements (“I watch what I eat to maintain my health” and “I watch what I eat to maintain a good appearance and prevent weight gain”) were answered on 5-point Likert scales (ranging from “always” (5) to “never” (1) for questions or from “I completely agree” (5) to “I do not agree at all” (1) for statements). After adding up the individual grades, a total sum was divided with maximum possible score (25) and an INA between 0 and 1 was formed for each participant. Such an approach has already been described in the literature for similar purposes [33].

In similar manner, the sustainability knowledge score (SKS) was calculated from one question on sustainable development and two statements on specific knowledge of fruit byproducts and edible films, which accounted for 52% of the total variance after factor analysis (Table 2). The question “Are you familiar with the term Sustainable Development?” was answered with “yes” (score 2) or “no” (score 1) while statements regarding edible films and fruit byproducts were answered using the 5-point Likert scales ranging from “I completely agree” (5) to “I do not agree at all” (1). After adding up the individual grades, a total sum was divided with a maximum score (12) that could be achieved, forming an SKS between 0 and 1 for each participant.

### 2.3. Data Analysis

All qualitative data were coded and evaluated using descriptive statistics with Stata and Microsoft Excel and analyzed.

Descriptive statistic, frequency distribution, and correlation analyses were performed to assess consumer behavior and attitude. Statistical differences between countries and generations were assessed using Kruskal–Wallis analysis of variance (ANOVA) with multiple comparison (two-tailed). Only variables that differed significantly between generations or countries were included in the principal component analysis (PCA). Data mining included classification and regression tree (C&RT), and an importance plot was generated using standard classification and regression tree after pruning on variance. Boxplots show median with 25 and 75 percentile and nonoutlier range. The answers obtained from all participants were also analyzed by means of multivariate analysis employing hierarchical cluster analysis using Ward’s method. The distance between samples was calculated using the square of the Euclidean distance with Minitab 17 statistical software. Statistica 14 (Tibco Software, Palo Alto, USA) was used for other data analysis, and a confidence level of *p* < 0.05 was considered significant.

## 3. Results and Discussion

### 3.1. Cookie Consumption and Preference Choice

**Figure 1:**
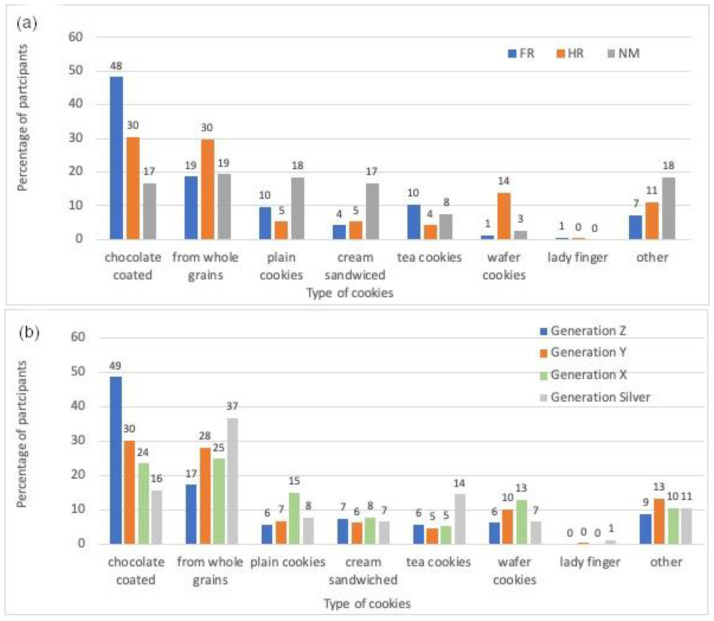
Preference (in percentage) of cookies type by (**a**) country of residence (FR = France; HR = Croatia; NM = North Macedonia) and (**b**) generation.

It is a well-known fact that cookies are, in general, a good source of energy, which 39% of the participants agreed with. In France and Croatia, cookies were consumed more frequently (one to five times per week) than in North Macedonia (one to two times per month) (*p* < 0.001). In addition, North Macedonians prefer whole-grain and plain cookies, while the French prefer chocolate-coated cookies and the Croats prefer both whole-grain and chocolate-coated cookies (Figure 1a). In agreement, Čukelj et al. [33] found that chocolate and whole-grain cookies were the most popular cookies among Croats.

Regarding the type of cookie, Generation Silver prefers whole-grain cookies, while the younger generations, especially Generation Z, prefer chocolate-coated cookies (Figure 1b). The assumption is that the younger generations value taste over nutritional value and healthy eating. This was also confirmed by responses to the question of whether they consider food to be one of the greatest pleasures, which Generation Z strongly agreed with, compared to the other generations, who partially agreed (median). Consistent with a previous study [34], it was found that Generation Z does care less about healthy eating and that they place less value on health advice. Overall, chocolate-coated cookies remain the most popular option, but Generations X and Silver tend to prefer whole-grain products due to their more health-conscious lifestyles.

Further findings highlight Generation Z as unique consumers compared to other generations. Generation Z answered in the affirmative more often (*p* = 0.021) than Generation Silver when asked if they preferred cookies to other sweets. Consumption of cookies as a morning or afternoon snack was reported more frequently by Generation Z (daily or three to five times per week) than by other generations (*p* < 0.001). While this reflects the nonideal diet of today’s generation, which is heavily influenced by the media, it is also possible that this generation lacks the cooking skills to prepare balanced meals or snacks themselves and relies on ready meals and available snacks from the store.

### 3.2. Nutritional Awareness and Interest in Health and Nutritional Value of Cookies

**Figure 2:**
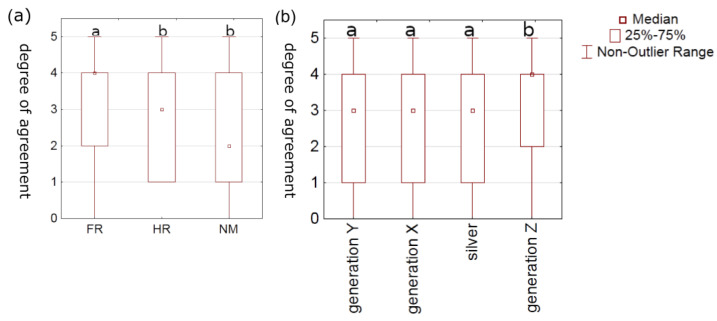
The agreement degree of consumers on the question whether they eat cookies out of habit, boredom, stress and/or other emotions depending on their (**a**) country of residence (FR = France; HR= Croatia; NM = North Macedonia) and (**b**) generation (1 = “I do not agree at all”; 5 = “I completely agree”). Boxplots with different letters represent statistical difference (*p* < 0.05) between countries or generations.

Compared to the other two nationalities, French participants reported eating cookies more often out of habit, boredom, or stress (*p* < 0.001) (Figure 2a). This may suggest that the French may be emotional consumers. Such findings are supported by the results of several studies; one of them examined the eating habits of elderly French people based on emotions and found that positive emotions were associated with higher food intake and vice versa [35]. Another reason could be that most of the French participants were students, for whom studies suggest that stress can play such a great role in their eating behavior that it can lead to eating disorders. [36]. When comparing generations, our result showed that Generation Z was more likely to consume cookies out of habit, boredom, or stress (*p* = 0.039) than the others (Figure 2b). Similar results were reported by Čukelj et al. [33], who showed a clear relation between habits and emotions. While it is not compelling that younger generations are more emotionally mature and resilient, our findings are in accordance with a study [37] that looked at stressful eating habits of Generation Z. Another study [38], which focused exclusively on the female Generation Z population, found mixed attitudes toward eating habits, with some females having eating disorders. Durukan and Gul [39] found that Generation Z’s eating habit discipline was significantly lower than that of previous generations, which is consistent with our data. Again, it is important to keep in mind that Generation Z was born in a time characterized by a variety of global issues. More recently, the COVID-19 pandemic also had profound effects on each generation, but Generation Z experienced more stress-related changes [40]. It should be noted that more detailed studies are needed to accurately determine the reason chosen by participants (habit vs. boredom vs. stress).

The majority of participants (86%) agreed that regular consumption of whole-grain products has a positive effect on their health. When asked if cookies are a good source of energy, opinions were divided. While some participants partially agreed (31%), others (32%) were undecided or disagreed (25%). Similarly, when asked if cookies are a good source of fiber, 28% of participants agreed, 30% could not decide, and 32.5% disagreed. In contrast to the Croats, the French did not rate the fiber content of cookies as very important (*p* < 0.001). This is in line with Lairon et al. [41], who reported that the overall intake of dietary fiber in the French population is insufficient. The reason could be that the French pay more attention to other aspects of cookies, such as sensory and hedonistic dimensions, or that they expect low sugar and calorie content.

**Figure 3:**
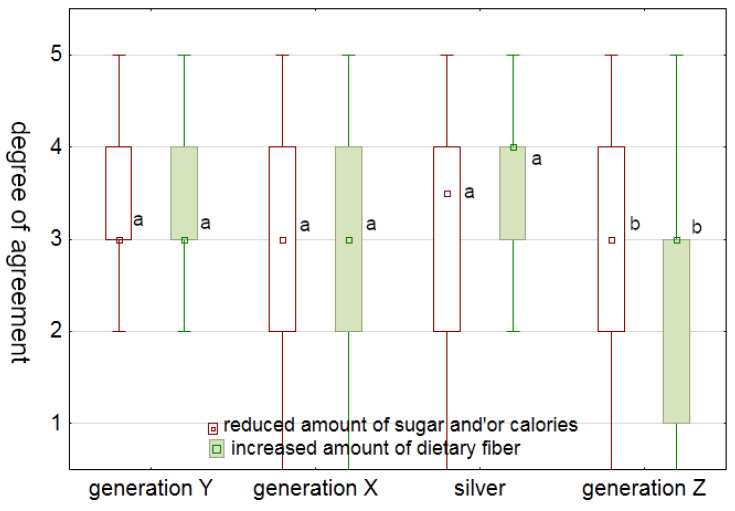
The importance that cookies have a reduced amount of sugar and/or calories or increased amount of dietary fiber to consumers of different generations (1 = “I do not agree at all”; 5 = “I completely agree”). Boxplots with different letters represent statistical difference (*p* < 0.05) within the same question between generations.

Unlike Generation Z, the participants of Generation Y agreed more (*p* = 0.0234) on the question “How important is it that cookies have less sugar and/or calories?” (Figure 3). This is supported by data showing that the majority of Generation Y responded positively when asked if they were aware of weight gain and therefore health. Similarly, Generation Z ranked the importance of cookies with higher fiber content lower than other generations (Figure 3). In terms of fiber consumption, we found a significant difference (*p* = 0.0002) between Generation Z, who reported consuming fiber most frequently (43%), three to five times per week, and Generation Y, who reported consuming fiber more frequently (38.5% of participants reported consuming daily and 42% of participants reported consuming it three to five times per week). An even larger difference (*p* < 0.001) was found between Generation Z (median = seldom) and the other generations (median = often) in terms of regular consumption of biologically active substances. It could be that Generation Z does not know exactly what biologically active substances or the related health benefits are. This could indicate that Generation Z generally lacks health education and health awareness. In contrast, a previous report [42] has shown that Generation Z is more health conscious than Generation Y. In another study, the health state of Generation Z was related to the successful health education that is received at a younger age [43].

Results indicate that Croatian and French consumers generally pay more attention to the ingredient list and labeling of cookies than North Macedonian consumers (*p* = 0.0007). The reason for this could be that nutrition labeling was only recently introduced in Northern Macedonia and people still need to learn how to read the labels. In addition, Generation Y reported reading food labels more often (always 34% and often 40%) than Generation Z (always 20% and often 34%). This suggests that there might be a lack of awareness due to inefficient health education for this generation. The lack of interest in health and misconceptions about appropriate nutrition might be caused by the influence of social media and advertisements.

**Figure 4:**
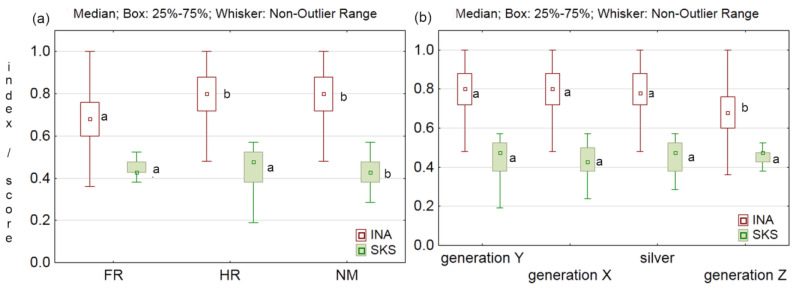
Nutritional awareness index (INA) and sustainability knowledge score (SKS) by (**a**) country of residence (FR = France; HR = Croatia; NM = North Macedonia) and (**b**) generation. Boxplots with different letters represent statistical difference (*p* < 0.05) within the INA or SKS between countries or generations.

Overall, the index of dietary awareness was significantly lower in French participants (0.67) compared to Croats (0.80) and Northern Macedonians (0.79) (Figure 4a). As expected, Generation Z has the lowest nutritional awareness compared to others, making them the main target group for health education (Figure 4b). Therefore, additional education is needed in the different population groups to improve nutrition knowledge and awareness.

### 3.3. Sustainability Knowledge Score (SKS)

Regardless of country or generation, the majority of participants (84%) were familiar with the importance of sustainable product development. In addition, some participants fully agreed (38%) or partially agreed (32%) that grape and aronia pomace are byproducts of the food industry rich in fiber and polyphenols, while some (26%) could not decide or disagreed (4%). Similarly, about half of the respondents agreed that edible films can extend shelf life (18% fully agreed, 32.5% partially agreed, while 41% neither agreed nor disagreed). This shows that knowledge about fruit byproducts and edible films is still insufficient. Differently from our study, Alonso and O’Neill [44] found that mature US consumers are more related to grape byproducts than the younger consumers group.

Our SKS depended significantly (*p* < 0.001) on the countries, with a median of 0.45 in France and 0.46 in Croatia, significantly different from 0.42 in Northern Macedonia (Figure 4a). Nevertheless, the consumer education level was the most important predictor of SKS. Based on the C&RT analysis, compared to the level of education, whose importance was 1, importance of the country of residence was 0.51, generation 0.38, and gender 0.27. Participants with a doctorate or master’s degree had a significantly (*p* < 0.001) higher SKS score (median 0.48) than participants with a bachelor’s degree, secondary or primary education (median 0.38). The lack of knowledge and understanding about food byproducts leads to a negative attitude towards their use [45]. Murillo et al. [46] showed that knowledge and previous consumption have a positive influence on the willingness to try food that contain seafood byproducts, whereas consumers’ concern about sensory quality, safety, and nutrition are main reasons for their avoidance. Sustainability knowledge could benefit consumers’ health and the economy of the food industry. Therefore, further education is needed on the potential use of fruit byproducts and edible films in the production of sustainable food.

### 3.4. Purchase Intention

Regardless of country of origin or generation, some participants (29%) showed interest in immediately purchasing new, attractive cookies from the market, but most were not interested. The majority of participants (79%) confirmed their interest in purchasing cookies with improved nutritional value that would positively impact their health, and most (67%) would be eager to pay a higher price for such cookies. In addition, about half of the participants report that they prefer whole-grain cookies (1) because they are good for their health (36% fully agreed and 15% partially agreed) and (2) because they provide a longer feeling of satiety (33% fully agreed and 12% partially agreed). The intention to purchase whole-grain cookies because they are good for health and because consuming cookies provides a longer feeling of fullness were intercorrelated (r = 0.76), and both intentions were also correlated with attitudes that it is important for cookies to have reduced sugar and/or calorie content (r = 0.61; 0.52), increased fiber content (r = 0.63; 0.62), and detailed information about cookies composition and nutritional value (r = 0.54; 0.53). Similarly, Čukelj et al. [33] found that participants with higher nutrition awareness were more likely to purchase the functional cookies. These findings suggest that further public education and promotion of the benefits of whole-grain products with a focus on food-based targets and messaging may be important to increase the consumption of whole grain cookies and thus the intake of dietary fiber [47].

**Figure 5:**
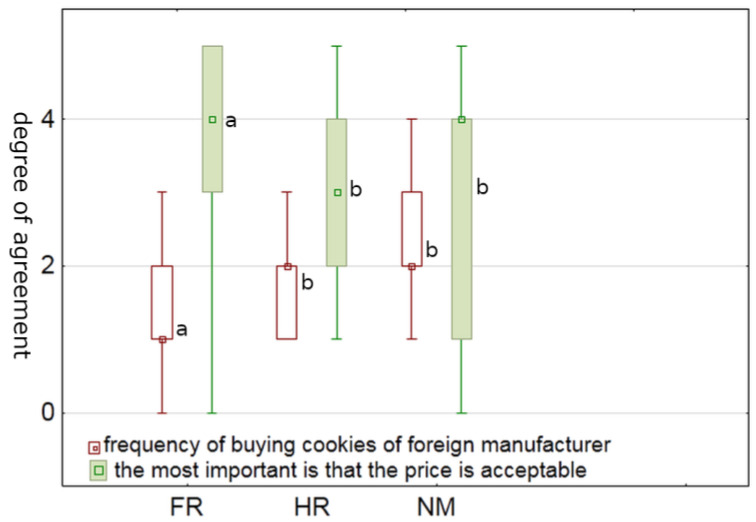
The influence of price and manufacturer on purchase intention by country of origin; FR = France, HR = Croatia, NM = North Macedonia (1 = never/I do not agree at all; 5 = always/I completely agree). Boxplots with different letters represent statistical difference (*p* < 0.05) within the same question between countries or generations.

France differed significantly from the other countries on several questions. When asked if participants would purchase foreign brands, the French were least likely to do so (Figure 5). Furthermore, compared to the other two countries (*p* = 0.0013), the French do not care about the manufacturer or brand as long as it is a product from France. This suggests that the French consider their own brands or the brand they usually consume or already know to be of higher quality and would prefer to purchase them. According to a survey by Statista (2021), French consumers expect brands to focus more on products made in France and French manufacturers [48].

In addition to brand choice, the price was also an important factor between countries (*p* < 0.001). In particular, the French agreed that an acceptable price was the most important factor (Figure 5). The results suggest that the most French population tends to be frugal when purchasing cookies. This makes sense given the turbulent changes that the French and global economies have undergone in recent years [49]. Recession, budget cuts, inflation, and stagnation in productivity all contribute to the struggling economy [50], leading consumers to purchase more affordable alternatives. However, compared to Croatia and Macedonia, France has a lower inflation rate and higher GDP, which indicate there might be other factors influencing this preference for more affordable cookies [51].

When looking at purchase intention by generation, Generation Y was more selective in choosing cookie brands and may have preferred imported cookies. Compared to the other generations, Generation Z seems to be more open-minded (*p* = 0.0199) when it comes to trying new types of cookies (Table S1 and Figure S1), but for them, a higher price was identified as a barrier to purchasing cookies. The fact that whole-grain cookies are good for health was not the key purchasing driver for Generation Z (Table S1), unlike the others (*p* < 0.001). This could be because Generation Z grew up in times of economic stress (i.e., high inflation) and is more concerned with pricing. The other generations are financially considerate, but not as much as Generation Z. This is also confirmed by evidence of greater frugality among the youngest generation [52]. A previous study of consumers in Kelantan [53] identified the key factors driving Generation Z to purchase snacks: packaging, price, available health information, availability, and taste.

**Figure 6:**
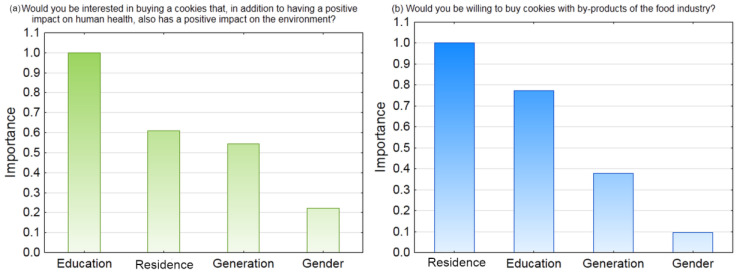
The importance plot of education level, country of residence, generation, and gender on consumer interest in purchasing cookies that (**a**) have a positive impact not only on human health but also on the environment and (**b**) contain byproducts of the food industry.

French and Croatian people were most likely to purchase cookies that had a positive environmental impact in addition to health benefits (median test: chi-square = 7.291, *p* = 0.026) (Table S2, Figure S1). Similarly, according to a study by Coderoni et al. [54], most participants prefer purchasing food enriched with byproducts that have lower environmental impact and promote health. Yet, the influence of the participants’ education level was a more important predictor of purchasing environmentally friendly cookies than the country of residence (Figure 6a).

Most participants were not enthusiastic about the idea of purchasing cookies containing food byproducts (Tables S1 and S2). This could be due to their lack of knowledge, shown by the SKS, and not knowing the health benefits of byproducts. Country of residence and education level were identified as the most important factors influencing the willingness to purchase cookies with food byproducts (Figure 6b and Figure S2). Among the three countries, French participants were the most interested in purchasing cookies with byproducts, while North Macedonians were the least interested (median test: chi-square = 74.208, *p* < 0.001). According to Yilmaz and Kahveci [20], generation, gender and commitment to recycling at home influences the willingness to purchase upcycled food. Consumers are more interested in purchasing products containing byproducts if they can expect a higher quality and better taste and if they believe that these products can contribute to solving the food waste problem [20]. Indeed, French consumers are more and more sensitive and have information about zero food waste and upcycling trends [55,56]. In contrast to our results, Grasso and Asioli [19] reported that although most British consumers had never heard of upcycled ingredients, they would be willing to purchase food products containing them. Our results suggest that investments should be made to increase knowledge about the benefits that food byproducts can provide, such as upcycled ingredients for environmental protection. In particular, Mediterranean countries with large production of grapes and other berries could benefit from using their byproducts.

### 3.5. Principal Components and Clusters

**Figure 7:**
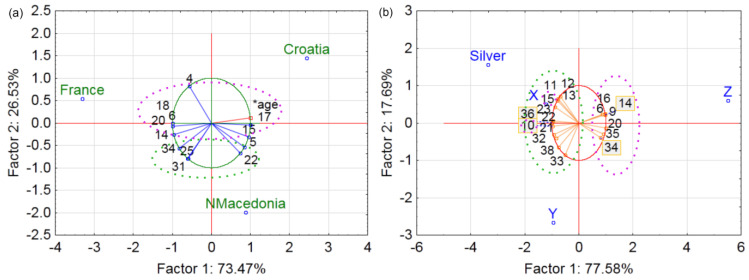
Principal component loadings and score plots of most significant questions by (**a**) country and (**b**) generation (Z, X, Y, and Silver). Legend: nutrition-related questions are circled with green dots (11—cookies are a good source of energy; 12—cookies can be a good source of dietary fiber; 13—eating cookies because they have fewer calories than some other sweets; 21—importance of reduced amount of sugar and/or calories when purchasing cookies; 22—importance of an increased amount of dietary fiber; 32—frequency of consumption of biologically active compounds; 33—frequency of consumption of dietary fiber; 36—eating to maintain my health; 37—reading nutritional values and ingredient list on food products; 38—index of nutrition awareness), consumption- and purchase-related questions are circled with pink dots (4—frequency of eating cereal-based products; 5—consumption of whole-grain products has a positive effect on health; 6—frequency of eating cookies; 9—eating cookies as a morning and/or afternoon snack; 10—eating cookies with coffee/tea; 14—eating cookies out of habit, boredom, stress, and/or other emotions; 15—favorite type of cookies; 16—purchasing cookies from a domestic producer; 17—purchasing cookies from a foreign producer; 18—manufacturer is not the key driver when purchasing cookies 20—importance of price when choosing cookies; 23—purchasing whole-grain cookies because they are good for health; 25—importance of having detailed nutritional information for cookies; 31—interest in purchasing cookies that have a positive effect not only on human health but also on the environment; 34—willingness to purchase cookies with byproducts from food industry; 35—eating is one of the greatest pleasures).

Principal component loadings (PCA) and score plots for questions with significant differences by country and generation are shown in Figure 7. PCA of the presented data by country extracted two components with eigenvalues of 8.82 and 3.18 that explained the complete variance. In terms of countries, North Macedonian consumers were more associated with the nutritional aspects of cookie consumption, whereas French and Croatian participants were more characterized by their consumption and purchase patterns. The greatest difference was found between French and North Macedonian participants, specifically in the type of cookies preferred, reasons for consumption, preference for purchasing cookies from domestic or foreign producers, interest in and willingness to purchase cookies with byproducts and/or nutritionally improved cookies (Figure 7a). While the French consume cookies more frequently, often out of boredom or stress, North Macedonians prefer chocolate-coated cookies and consume them less frequently, as discussed in Section 3.1. In addition, the French prefer domestic producers and are most interested in purchasing cookies with byproducts that have a positive impact on the environment and people’s health, while Macedonians are more open to foreign producers, as described in Section 3.4.

PCA of the presented data between generations (Figure 7b) explained that the first two components accounted for 95.27% of the total variance, with eigenvalues of 14.74 and 3.36, respectively. Whereas Generation Z was associated with the consumption and purchase habits, more mature generations were more concerned with the nutritional aspects. The largest difference found between generations Y and Z can be attributed to the type of cookies consumed (with coffee/tea or as an afternoon snack), the reasons for consumption, the consumption of bioactive compounds and dietary fiber, and reading labels.

**Figure 8:**
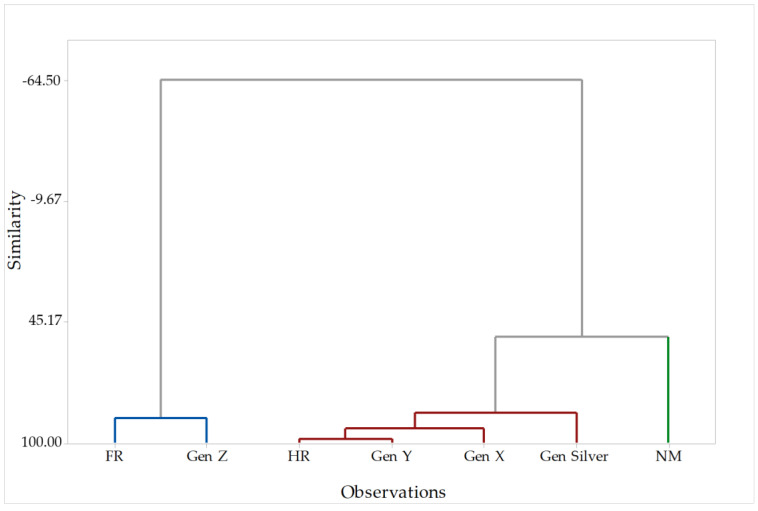
Cluster diagram of the answers (observations) of all participants depending on the country of origin (FR = Franc; HR = Croatia; NM = North Macedonia) and generation (GEN). Different colors are needed as they indicate different clusters in cluster analysis.

Cluster analysis was also performed to determine the similarity between countries and generations (Figure 8). The data can be divided into two main groups consisting of three clusters: blue, red, and green. The first group, as well as the blue cluster, represents France and proves that most of the participants belong to Generation Z. This group differs from the second group, consisting of the red and green cluster representing Croatia and North Macedonia. The differences between France and North Macedonia were already noted in the PCA. It was also shown that the participants in France tend to be emotional eaters, while Croats and North Macedonians tend to have similar eating habits and cookie preferences, thus belonging to the same group. This is not surprising, since most of the French participants were part of the student population, unlike participants from the other two countries. Moreover, Croatia and Northern Macedonia are geographically closer (Balkan Peninsula) and were part of the same country in the past (former Yugoslavia). On the other hand, the participants from North Macedonia provided contrary answers to some of the questions, which is why they form a separate group. As for the second group, the number of participants was more evenly distributed among generations, especially in Northern Macedonia, while the Croatian population was slightly dominated by Generation Y.

### 3.6. Limitations of Study

The main limitation of this study is that most of the study participants were female, which significantly affects its representativeness. On the other hand, women are responsible for purchasing in most households. Another limitation is the uneven number of participating populations from France, Northern Macedonia, and Croatia as well as the small number of participants and uneven distribution between generations. Nevertheless, our results could serve as a guide for the development of new products and marketing strategies for healthier and more sustainable cookies.

## 4. Conclusions

This study investigated the market potential and consumer attitudes towards whole-grain cookies with food byproducts and edible films in Croatian, French, and Northern Macedonian populations. Our study confirms that cookies are a popular food that many consumers eat regularly. Whole-grain cookies are among the most preferred types of cookies, which reflects consumer nutritional awareness. Consumer attitudes and consumption habits vary by country and generation, with Generation Z differing from other generations. The majority of consumers are familiar with sustainability and interested in purchasing cookies that are beneficial to the environment and their health. However, their knowledge of fruit byproducts or edible films and their willingness to purchase cookies with food byproducts is limited. Additional educational programs are needed for all generations to raise awareness of the opportunities that upcycled byproducts or edible films offer for environmental protection but also human health. Hence, the findings of this study could be used to develop a marketing and education strategies for communicating the benefits of using upcycled ingredients in producing food of enhanced nutrition value. Such strategies could result in consumers’ better acceptability of food with upcycled ingredients and thus a rise in circular economy. In particular, Mediterranean countries with big production of grapes and related byproducts could benefit from such strategies. Future studies should explore the relevance of various educational programs to upcycling food byproducts.

## Figures and Tables

**Table 1:** Participants’ characteristics for the total sample and according to gender (numbers of participants with percentages in brackets).

Sociodemographic Characteristic				
	Overall (*n* = 757)	Croatia (*n* = 472)	France (*n* = 166)	North Macedonia (*n* = 119)
Gender ^1^, *n* (%)				
Female	621 (82)	389 (82)	133 (80)	99 (83)
Male	135 (18)	83 (18)	32 (19.3)	20 (17)
Age cohorts, *n* (%)				
Generation Z	191 (25)	58 (12)	116 (70)	17 (14)
Generation Y	337 (45)	278 (59)	17 (10)	42 (35)
Generation X	153 (20)	88 (19)	20 (12)	45 (38)
Generation Silver	76 (10)	48 (10)	13 (8)	15 (13)
Highest education qualification, *n* (%)				
Primary school	4 (0.5)	3 (0.6)	1 (0.6)	/
Secondary school	87 (11.5)	74 (16)	6 (3.6)	7 (6)
Bachelor’s or master’s	497 (66)	276 (58.4)	109 (65.8)	112 (94)
PhD	169 (22)	119 (25)	50 (30)	/
Employment status, *n* (%)				
Employed, full- or parttime	503 (66.4)	381 (81)	20 (12)	102 (86)
Student	154 (20.3)	30 (6.4)	114 (69)	10 (8)
Entrepreneur	30 (4)	30 (6.4)	/	/
Retired	10 (1.3)	5 (1.1)	1 (0.6)	2 (2)
Other	60 (8)	24 (5.1)	31 (18.4)	5 (4)

^1^ Participant (0.1%) did not want to report their gender.

**Table 2:** Contribution of questions to the index of nutrition awareness and sustainability knowledge score.

Index of Nutrition Awareness	Component
Do you introduce biologically active compounds into your body daily?	0.608
How often do you consume food rich in dietary fiber?	0.713
To what extent do you agree with the following statement: “I watch what I eat to maintain my health”?	0.765
How much do you agree with the following statement: “I watch what I eat to maintain a good appearance and prevent weight gain”?	0.644
Are you reading food labels; particularly nutritional values and ingredient list on food products?	0.615
Explanation of variance	45%
Eigenvalue	2.257
Sustainability knowledge score	
Are you familiar with the term Sustainable Development?	0.533
How much do you agree with the following statement: “Edible films are active packaging systems that extend product shelf life, improve product quality, and contribute to the nutritional quality of the final product”?	−0.798
To what extent do you agree with the following statement: “Grape and/or aronia pomace is a byproduct of the food industry rich in dietary fiber and polyphenols”?	−0.806
Explanation of variance	52%
Eigenvalue	1.57

## Data Availability

The data generated or analyzed during this study are available from the corresponding author upon reasonable request.

## Supplementary Materials

The following supporting information can be downloaded at: https://www.mdpi.com/article/10.3390/foods12213932/s1. Table S1: Purchase intention of whole-grain, nutritionally improved and sustainable cookies by generations (number with percentage in brackets). Table S2: Purchase intention of whole-grain, nutritionally improved, and sustainable cookies by country (number with percentage in brackets); Figure S1: Classification and regression tree of willingness to buy environmentally friendly cookies; Figure S2: Classification and regression tree of willingness to buy cookies with food byproducts.
