# Supplementary material for: Consumer Nutritional Awareness, Sustainability Knowledge, and Purchase Intention of Environmentally Friendly Cookies in Croatia, France, and North Macedonia
Source: Foods. 2023 Oct 27;12(21):3932. doi: 10.3390/foods12213932 (PMC10650571; doi:10.3390/foods12213932)
Supplement: Supplementary file 1 [file foods-12-03932-s001.zip › foods-2665897-supplementary.pdf]

Table S1. Purchase intention of whole grain, nutritionally improved and sustainable cookies by generations (number with percentage in brackets).

	Generation Z, n = 191	Generation Y, n = 337	Generation X, n = 153	Silver Generation, n = 76
I buy whole grain cookies because they are good for health				
I completely agree	16 (8)	49 (14)	25 (16)	23 (30)
I partially agree	57 (30)	129 (38)	62 (40)	25 (33)
I neither agree nor disagree	51 (27)	100 (30)	28 (18)	18 (24)
I somewhat disagree	22 (12)	21 (6)	10 (7)	2 (3)
I do not agree at all	31 (16)	29 (9)	18 (12)	7 (9)
Did not answer	14 (7)	9 (3)	10 (7)	1 (1)
Would you be attracted to a cookie with a changed or improved nutritional value (e.g., less sugar and/or fat, added minerals, added fiber, added antioxidants) if you knew that it had a beneficial effect on your health?				
Yes	155 (81)	266 (79)	119 (78)	61 (80.3)
No	24 (13)	62 (18)	26 (17)	14 (18.4)
Did not answer	12 (6)	9 (3)	8 (5)	1 (1.3)
Would you be willing to pay a higher price for cookies with altered or improved nutritional value (compared to a conventional cookie)?				
Yes	107 (56)	243 (72)	104 (68)	51 (67.1)
No	72 (38)	86 (26)	41 (27)	24 (31.6)
Did not answer	12 (6)	8 (2)	8 (5)	1 (1.3)
Would you be interested in buying cookies that, in addition to having a positive impact on human health, also has a positive impact on the environment?				
Yes	184 (96)	317 (94)	145 (95)	73 (96)
No	7 (4)	20 (6)	8 (5)	3 (4)
Would you be willing to buy cookies with by-products of the food industry?				
Yes	152 (80)	252 (75)	96 (63)	50 (66)
No	39 (20)	85 (25)	57 (37)	26 (34)

Table S2. Purchase intention of whole grain, nutritionally improved and sustainable products by country (number with percentage in brackets).

	Croatia, n = 472	France, n = 166	North Macedonia, n = 119
I buy whole grain cookies because they are good for health			
I completely agree	77 (16)	18 (11)	18 (15)
I partially agree	189 (40)	46 (28)	38 (32)
I neither agree nor disagree	123 (26)	48 (29)	26 (22)
I somewhat disagree	35 (7.5)	17 (10)	3 (2.5)
I do not agree at all	48 (10.5)	25 (15)	12 (10)
Did not answer		12 (7)	22 (18.5)
Would you be attracted to a cookie with a changed or improved nutritional value (e.g. less sugar and/or fat, added minerals, added fiber, added antioxidants) if you knew that it had a beneficial effect on your health?			
Yes	385 (82)	129 (78)	87 (73)
No	87 (18)	26 (15.5)	13 (11)
Did not answer		11 (6.5)	19 (16)
Would you be willing to pay a higher price for cookies with altered or improved nutritional value (compared to a standard cookie)?			
Yes	352 (75)	78 (47)	75 (63)
No	120 (25)	78 (47)	25 (21)
Did not answer		10 (6)	19 (16)
Would you be interested in buying cookies that, in addition to having a positive impact on human health, also has a positive impact on the environment?			
Yes	453 (96)[a]	158 (95)[a]	108 (91)[b]
No	19 (4)[a]	8 (5)[a]	11 (9)[b]
Would you be willing to buy cookies with by-products of the food industry?			
Yes	361 (76) [a]	140 (84) [a]	49 (41) [b]
No	111 (24) [a]	26 (16) [a]	70 (59) [b]

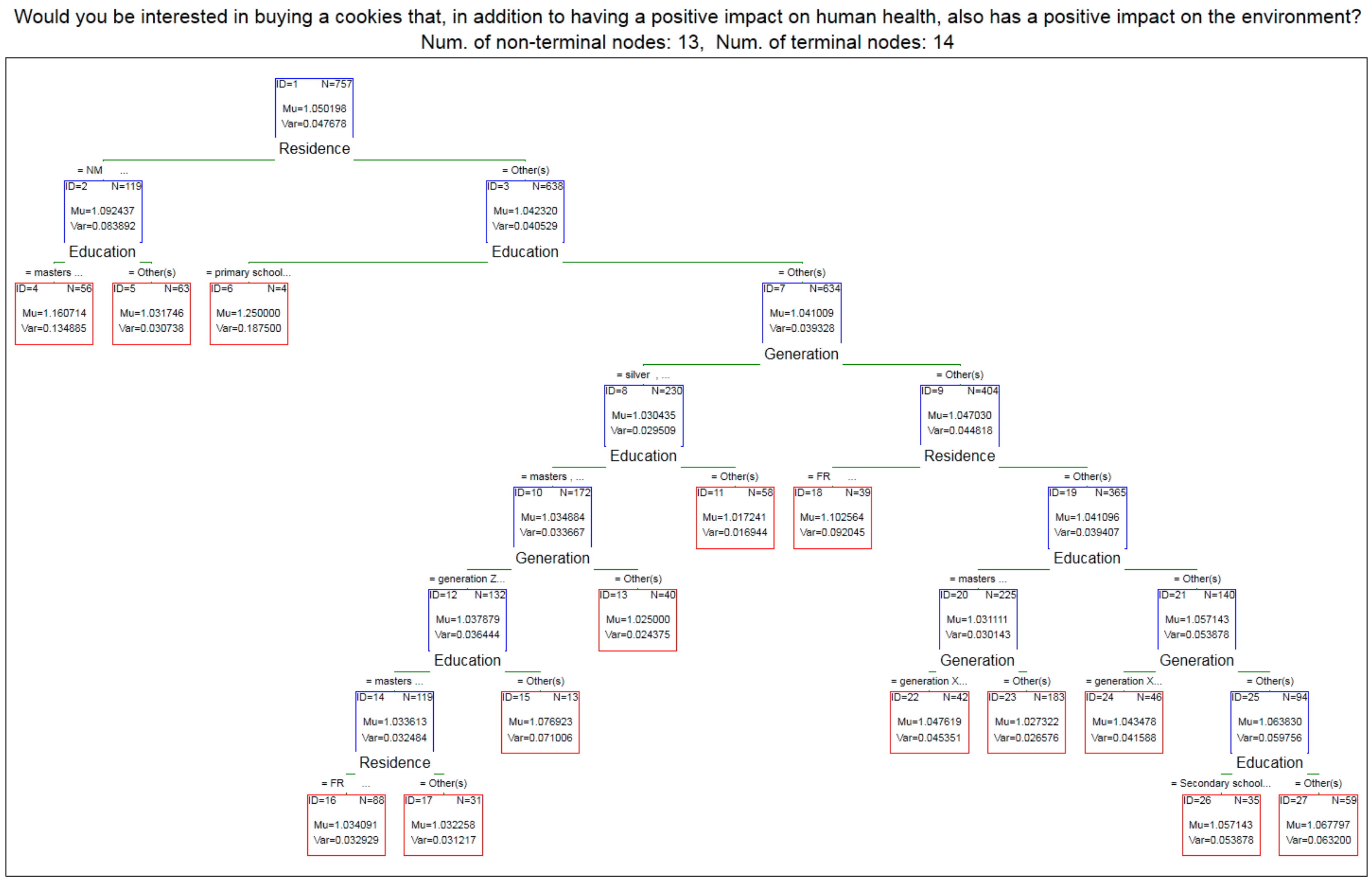

Figure S1. The classification and regression tree on the consumers interest in buying cookies that in addition to having a positive impact on human health, also have a positive impact on the environment.

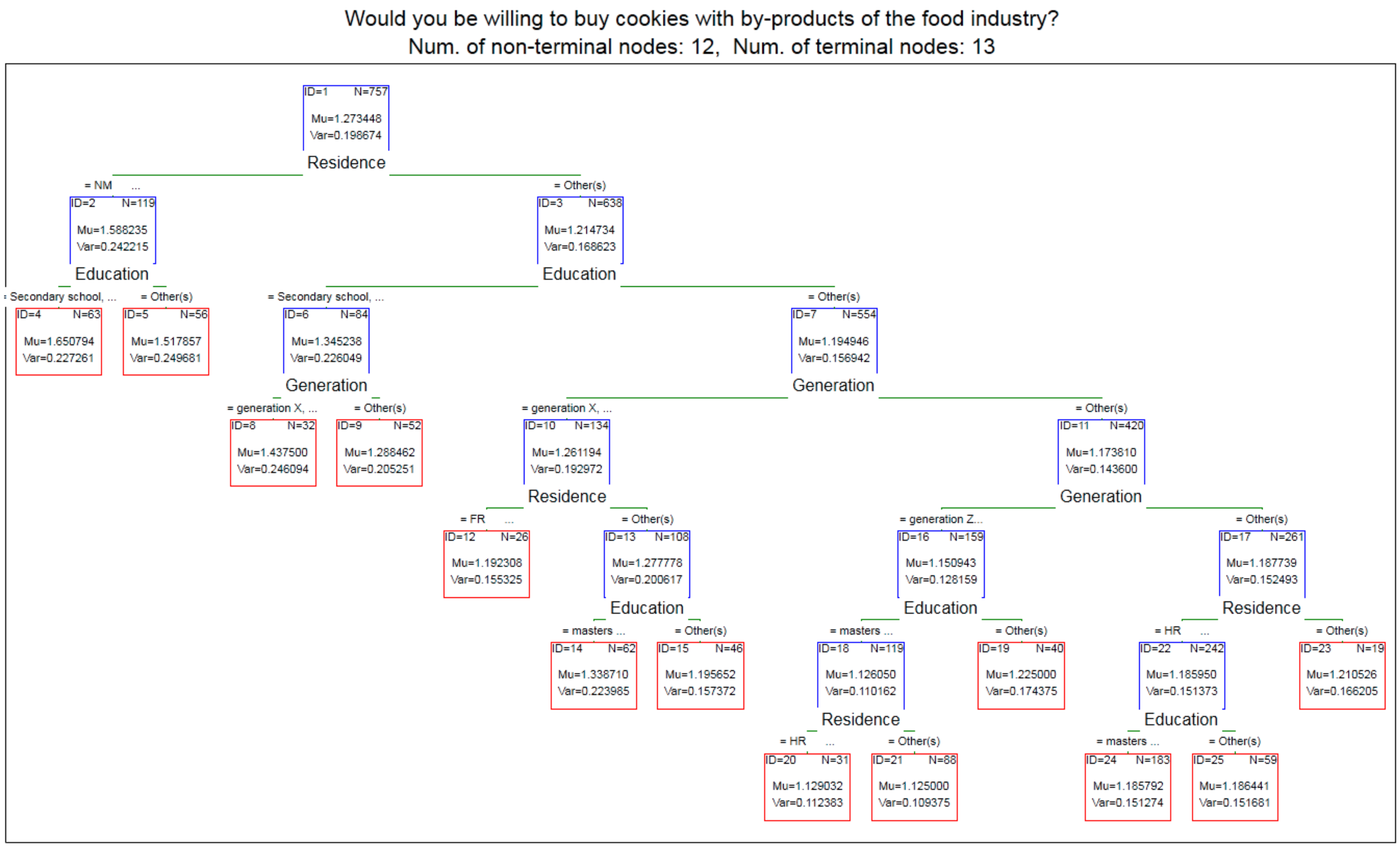

Figure S2. The classification and regression tree on the consumers' willingness to buy cookies with by-products of food industry.
